# Efficient single-channel current measurements of the human BK channel using a liposome-immobilized gold probe

**DOI:** 10.1007/s44211-024-00707-3

**Published:** 2024-12-20

**Authors:** Minako Hirano, Mami Asakura, Toru Ide

**Affiliations:** Graduate School of Interdisciplinary Science and Engineering in Health Systems, Okayama University, 3-1-1 Tsushima-naka, Kita-ku, Okayama-shi, Okayama, 700-8530 Japan

**Keywords:** Human BK channel, Artificial lipid bilayer recording, Ion channel current, Single-channel recording

## Abstract

The human BK channel (hBK) is an essential membrane protein that regulates various biological functions, and its dysfunction leads to serious diseases. Understanding the biophysical properties of hBK channels is crucial for drug development. Artificial lipid bilayer recording is used to measure biophysical properties at the single-channel level. However, this technique is time-consuming and complicated; thus, its measurement efficiency is very low. Previously, we developed a novel technique to improve the measurement efficiency by rapidly forming lipid bilayer membranes and incorporating ion channels into the membrane using a hydrophilically modified gold probe. To further improve our technique for application to the hBK channel, we combined it using the gold probe with a liposome fusion method. Using a probe on which liposomes containing hBK channels were immobilized, the channels were efficiently incorporated into the lipid bilayer membrane, and the measured channel currents showed the current characteristics of the hBK channel. This technique will be useful for the efficient measurements of the channel properties of hBK and other biologically important channels.

**Graphical abstract:**

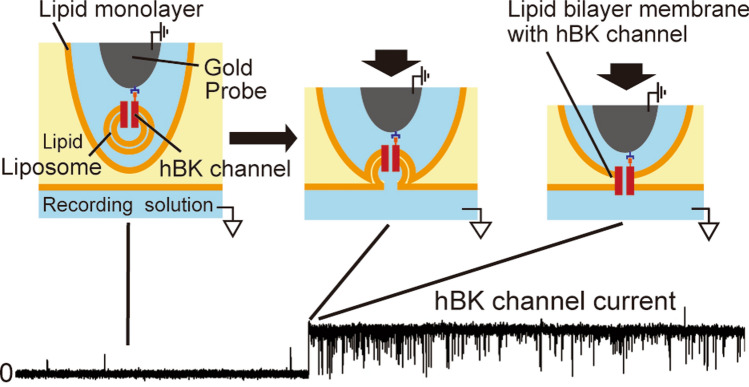

**Supplementary Information:**

The online version contains supplementary material available at 10.1007/s44211-024-00707-3.

## Introduction

Ion channels are crucial membrane proteins that regulate a wide range of biological functions by controlling ion flow in response to stimuli [1–3]. Due to their importance, dysfunction of ion channels causes serious diseases [4]. Therefore, it is important to identify and develop drugs that target ion channels [4].

A large-conductance, voltage, and calcium-activated potassium channel (BK channel) is a crucial membrane protein that regulates a wide range of biological functions, such as neurotransmitter release, muscle relaxation, or hormone release, by controlling the flow of ions in response to stimuli [2, 5–8]. Mutations in BK channels are associated with epilepsy and hypertension [2, 5, 9, 10].

To identify drugs that act on the human BK channel (hBK channel), it is important to understand the biophysical properties of the channel, such as its gating properties and ligand binding sites. Single-channel recording is the best method for understanding channel properties. This method allows us to measure ionic currents through ion channels at the single-channel level. Artificial lipid bilayer recording is a technique to measure channel currents through ion channels reconstituted in an artificial bilayer membrane at the single-channel level [11]. This method can be used to measure channel properties under various membrane lipid compositions and solution conditions. This allows the effect of the drug candidates on the target channels to be studied in detail. However, the measurement efficiency is very low because the process of preparing an artificial membrane and incorporating channels into the membrane is time-consuming and complicated [11, 12].

Many attempts have been made to improve the measurement efficiency. Several techniques have been developed to produce multiple bilayer membranes simultaneously in a short period. Several studies have reported that, by contacting two recording solutions or hydrogels containing a recording solution in a lipid solution, a lipid bilayer membrane is instantaneously formed at the interface [13–18].

In addition, we developed techniques to promptly incorporate ion channels into the bilayer membrane simultaneously with the membrane formation. By contacting an ion channel-immobilized support substrate (hydrogel beads or polyethylene glycol (PEG)-coated gold probes) with a recording solution in a lipid solution, a lipid bilayer membrane is formed on the substrate surface and the ion channels are simultaneously incorporated [18–23]. Using this technique, channel currents can be detected within a few minutes, whereas a conventional artificial lipid bilayer recording technique requires tens of minutes or hours. We have reported that the channel currents of several types of ion channels can be measured using these techniques; in particular, one of the potassium channels, the KcsA channel, is easily and repeatedly incorporated into the membrane [20]. However, it is difficult to measure the currents of certain ion channels, including the hBK channel.

Here, we improved our techniques to efficiently measure the channel currents of the hBK channels. This technique could potentially be applied to other ion channels that are difficult to incorporate into bilayer membranes.

## Materials and methods

### hBK channel expression and purification

The α-subunit of the hBK channel protein with a histidine tag (His-tag) was expressed in an insect cell (Sf9) using the Bac-to-Bac expression system (Thermo Fisher Scientific, USA) and purified using Co^2+^ affinity gel beads as previously described with some modifications [16]. Briefly, the hBK channel gene with a C-terminal His-tag was introduced into a baculovirus shuttle vector (bacmid). Recombinant baculovirus was obtained by transfecting Sf9 cells with recombinant bacmid DNA. Sf9 cells were infected with recombinant baculovirus in flasks at 27 °C for 48 h to express the hBK channel protein. Infected cells were sonicated, and the membrane fraction was solubilized with 2% Triton X-100. The extracted hBK channel protein was purified using Co^2+^ affinity gel beads (TALON Metal Affinity Resin, TaKaRa, Japan) and then loaded onto a HiPrep 16/60 Sephacryl S-300 HR column (Cytiva, USA) to isolate hBK channels and replace the buffer with BK buffer (50 mM sodium phosphate, 10 mM KCl, 10 mM NaCl, 5 mM *n*-dodecyl-β-D-maltoside (DDM), protease inhibitors (pH 7.0)).

### Gold probes on which surfactant-solubilized hBK channels were immobilized (“Probe-hBK”) or liposomes containing hBK channels were immobilized (“Probe-hBK-Liposome”)

**Fig. 1:**
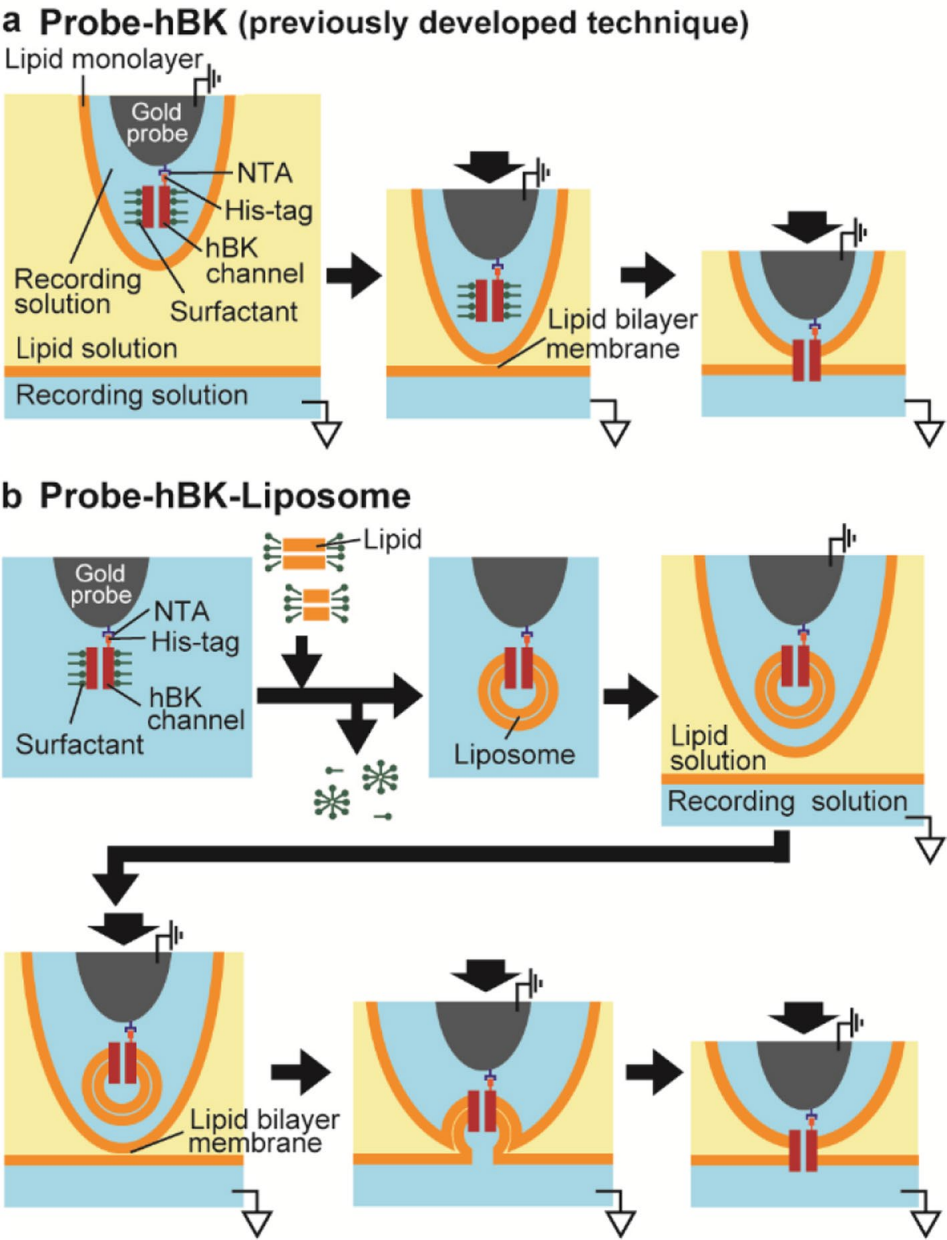
Schematic illustration of ion channel current recording techniques using a gold probe. **a** Our previously developed technique. The probe was hydrophobically modified, and surfactant-solubilized hBK channels were immobilized on the probe (the probe is called “Probe-hBK”). When the Probe-hBK was moved to a recording solution through a lipid solution and touched the interface between them, a lipid bilayer membrane was formed, and then the immobilized hBK channels were incorporated into the membrane. **b** The novel technique using a liposome. When the Probe-hBK was immersed in a surfactant-solubilized lipid solution and the surfactants were removed by BIO-BEADs, the immobilized hBK channels were reconstituted into liposomes (“Probe-hBK-Liposome”). When the Probe-hBK-Liposome was contacted between the recording solution and the lipid solution, the bilayer membrane was formed, and the liposomes were fused into the membrane

We chemically modified the tip of a gold probe (contact probe, KS-100 305 090 A 2000, INGUN, Germany) to cover it with a PEG layer containing nitrilotriacetic acid (NTA) chelated to Ni^2+^ in addition to a hydrophobic area, as previously reported [23]. To prepare the Probe-hBK, the modified probe tip was then incubated for 15 min in a solution of 0.10–0.28 µg/ml hBK channel proteins with the His-tag to immobilize the channels on the surface of the probe (Fig. 1a). To prepare the Probe-hBK-Liposome, the Probe-hBK was then immersed in 500 µl of a surfactant-solubilized lipid solution (0.45 mg/ml 1-palmitoyl-2-oleoyl-glycero-3-phosphocholine (POPC), 1.8 mM *n*-decyl-β-D-maltoside (DM), 200 mM KCl, 10 mM Tris–HCl (pH7.0), 4.0 µM CaCl_2_) for more than 2 h. About 100 µg of BIO-BEADs (Bio-Rad, USA) were then added to the solution and mixed thoroughly to rapidly remove surfactants and reconstitute liposomes containing hBK channels (Fig. 1b). The probe was washed with recording buffer (200 mM KCl, 10 mM Tris–HCl (pH7.0), 4.0 µM CaCl_2_) immediately before recording channel currents.

### Formation of the lipid bilayer membrane containing the hBK channels

The Probe-hBK or Probe-hBK-Liposome was moved into a chamber containing the recording solution (200 mM KCl, 10 mM Tris–HCl (pH7.0), 4.0 µM CaCl_2_) with a layer of a lipid solution (30 mg/ml asolectin extracted soybeans (Sigma-Aldrich, USA) in *n*-decane) (Fig. 1). When the probe touched the interface between the lipid solution and the recording solution, a lipid bilayer membrane was formed. At that time, when hBK channels were reconstituted in the membrane, channel currents appeared.

### Ionic current recording

Ionic currents were recorded as previously described [23]. In this method, the gold probe was held at the virtual ground, and an electrode in the recording solution was connected to a patch-clamp amplifier so that all membrane potentials indicated the potential in the recording solution.

## Results and discussion

**Fig. 2:**
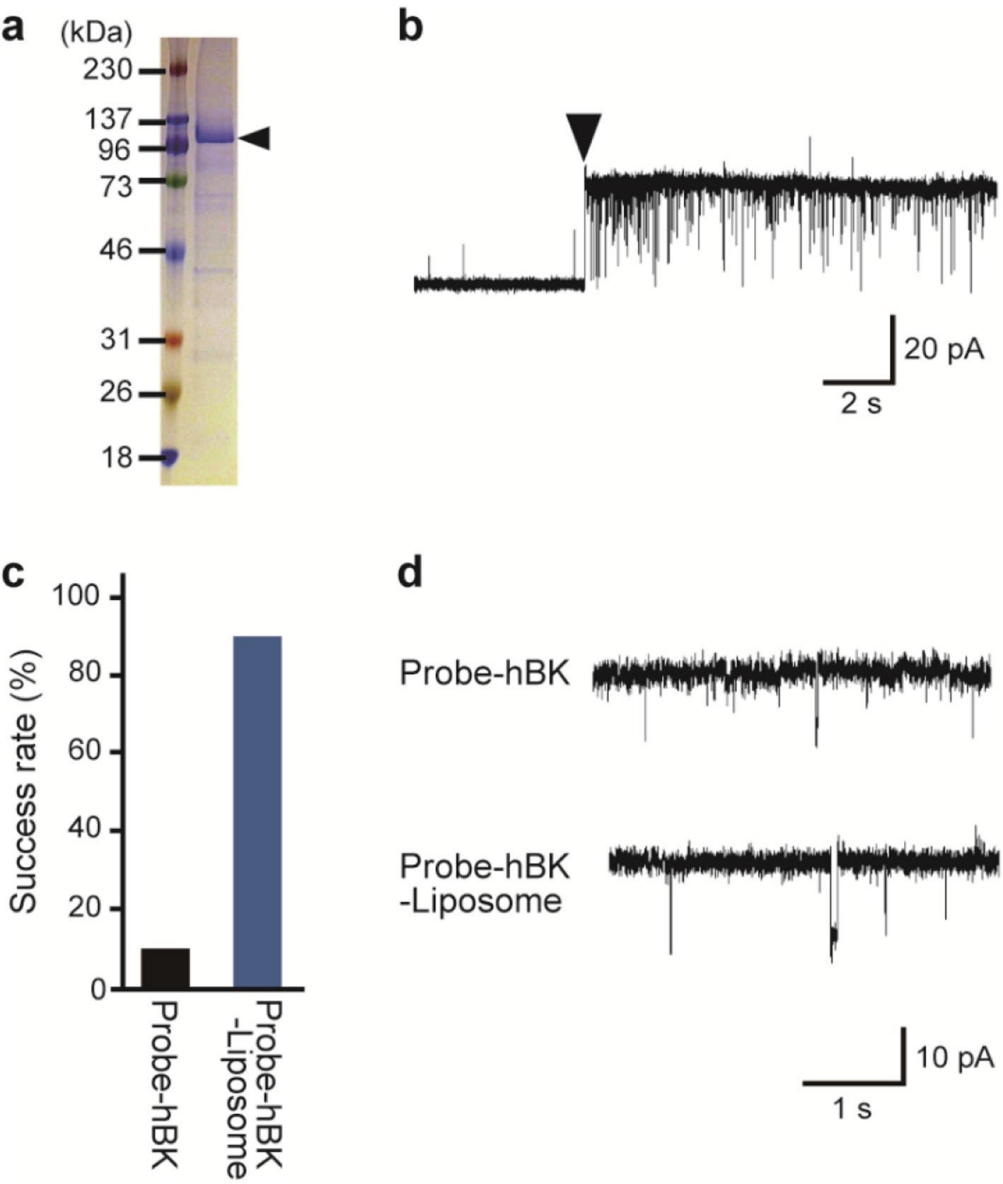
Improvement of the efficiency of single-channel current measurement using the probe on which liposomes containing hBK channels were immobilized (“Probe-hBK-Liposome”). **a** SDS-PAGE image of purified hBK channel. **b** Typical time course of current measured at + 140 mV when the Probe -hBK-Liposome contacted the interface between the lipid solution and the recording solution. The hBK channel was reconstituted in the lipid bilayer membrane (▼). **c** Success rate of channel current measurements using the probe with solubilized hBK channels (“Probe-hBK”) or the Probe-hBK-Liposome. **d** Typical channel current traces of the hBK channel measured using the Probe-hBK and the Probe-hBK-Liposome at + 60 mV

The hBK channel protein with the His-tag was expressed in insect cells and purified via affinity chromatography and gel filtration chromatography. The purified proteins were confirmed by SDS-PAGE, and a major band was detected between the 96 kDa and 137 kDa marker proteins (Fig. 2a). This molecular weight was similar to the expected molecular weight of the hBK channel protein (approximately 125 kDa).

We prepared two types of gold probes, the probe on which surfactant-solubilized hBK channels were immobilized (“Probe-hBK”) and the probe on which liposomes containing hBK channels were immobilized (“Probe-hBK-Liposome”). The former probe, which we have previously reported [20, 23], was modified with PEG to cover the surface with an aqueous solution, and the surfactant-solubilized ion channels were immobilized on the PEG layer via His-tags at the C-terminus (Fig. 1a). The Probe-hBK-Liposome was prepared as follows (Fig. 1b). The Probe-hBK was immersed in a surfactant-solubilized lipid solution to replace surfactants with lipids. Liposomes were then formed around the channels by removing the surfactants bound to lipids using BIOBEADs.

Channel currents were observed by moving the probe through a lipid solution into a recording solution and by contacting the probe with the interface between them (Figs. 1, 2b). A lipid bilayer membrane was formed on the surface of the probe at the interface. When the Probe-hBK was used, the immobilized channels were directly incorporated into the membrane. In the case of the Probe-hBK-Liposome, hBK channels were incorporated into the membrane by fusing liposomes containing hBK channels.

**Fig. 3:**
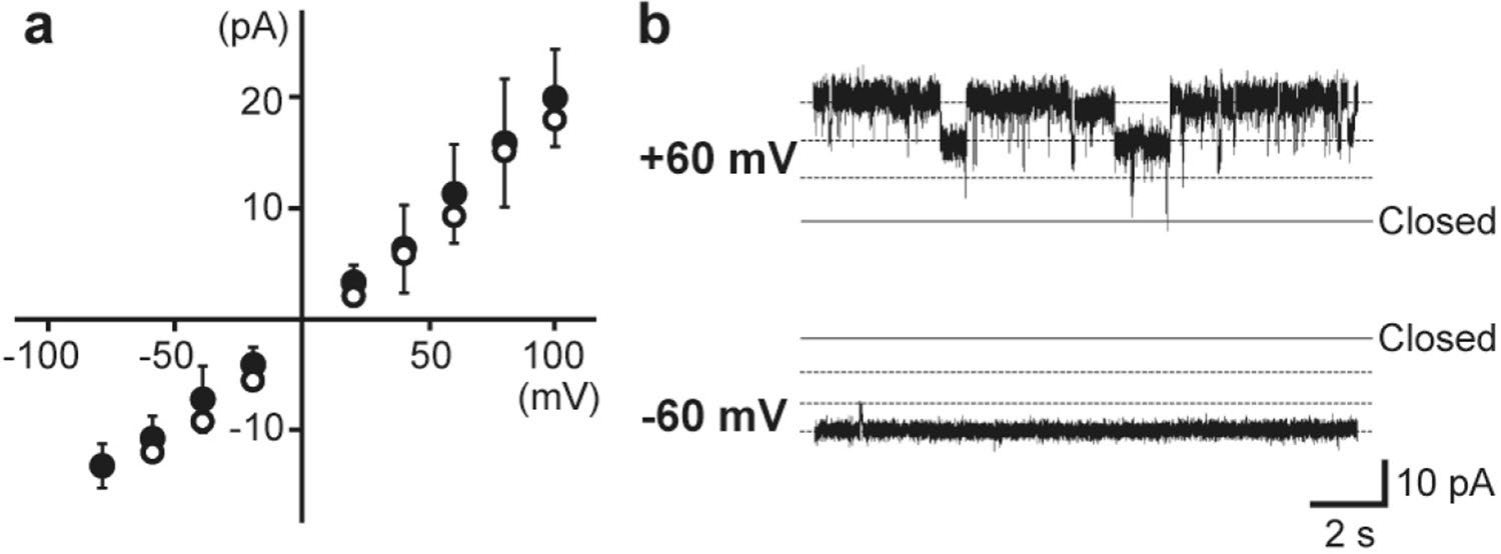
Single-channel current recordings of the hBK channel measured using the probe with hBK channel-containing liposomes (“Probe-hBK-Liposome”). **a** A current–voltage (I–V) relationship for the hBK channel measured using the Probe-hBK-Liposome (black circle (●), n = 3–8). The open circles (○) indicate a representative I–V relationship measured using the probe with solubilized hBK channels (“Probe-hBK”). **b** Typical channel current traces of the hBK channel at + 60 mV and − 60 mV

The hBK channel current was measured more efficiently with the Probe-hBK-Liposome than with the Probe-hBK. We observed hBK channel currents in 90% of the measurements using the Probe-hBK-Liposome (Fig. 2b, c, n = 10), whereas we detected channel currents in only 10% of the measurements using the Probe-hBK (Fig. 2c, n = 10). The current traces observed using the Probe-hBK and the Probe-hBK-Liposome were nearly identical; both had noise levels of approximately 2 pA, and their current–voltage (I-V) relationships were also almost the same (Figs. 2d, 3a, n = 10). These results indicate that the hBK channel could be more easily incorporated into the membrane using the Probe-hBK-Liposome than using the Probe-hBK. This may be because liposome fusion occurs easily with the Probe-hBK-Liposome, similar to the liposome fusion method used in the conventional artificial bilayer technique.

Measurement of the hBK channel current using the Probe-hBK was difficult, although we reported that the KcsA channel current could be measured with high efficiency using a probe on which KcsA channels were immobilized [20]. This difference could be attributed to the number of transmembrane regions. The KcsA channel has only two transmembrane regions in each subunit, whereas the hBK channel has seven transmembrane regions. As more surfactants bind to the transmembrane region of the BK channel than to that of the KcsA channel, it may be more difficult to replace surfactants with lipids in the hBK channels. In the Probe-hBK-Liposome, surfactants bound to the hBK channels on the probe were removed beforehand, and the hBK channels were reconstituted into liposomes, with the transmembrane region of the channel covered by lipids. Since liposomes spontaneously fuse with the lipid bilayer membrane, the hBK channels on the Probe-hBK-Liposome were incorporated into the membrane by liposome-membrane fusion. This process enabled efficient measurement of the hBK channel currents.

The hBK channel currents measured with the Probe-hBK Liposome were observed at a single-channel level. Single-, double-, and triple-channel currents were detected in 30%, 30%, and 20% of measurements, respectively, and no more than six channels were detected (n = 9). Therefore, we were able to determine the channel properties at a single channel level. Additionally, all channel currents measured with the Probe-hBK Liposome were recorded for at least 1 min, with 78% of the currents measured for more than 5 min (n = 9), which is sufficient to investigate the effect of drugs on the hBK channel.

The success rate of the hBK channel current measurement using the Probe-hBK-Liposome gradually decreased when the same probe was used repeatedly. After contacting the probe with the interface between the lipid solution and the recording solution and confirming the hBK channel current, the probe was separated from the interface and then contacted again at the interface. This procedure was repeated four times. The success rates of the hBK channel current measurement using the probe for two, three, and four times were 44%, 11%, and 0%, respectively (n = 9). The channel current traces and I-V relationships measured in each measurement were nearly identical (Fig. S1). This decrease in the success rate could be caused by the detachment of some hBK channels from the probe upon contact with the lipid bilayer. Therefore, to efficiently measure the hBK channel current, it is necessary to change the Probe-hBK-Liposome for each measurement.

Figure 3 shows the properties of hBK channels in the presence of 4 μM CaCl_2_. As the His-tag is located on the intracellular side of the hBK channel, its intracellular region is on the side of the probe. Because Ca^2+^ binding to the binding region of the intracellular domain opens the hBK channel, the probe was immersed in a solution containing CaCl_2_ prior to bilayer formation. The single channel I–V relationship was linear (Fig. 3a), and the conductance was 193.9 ± 39.6 pS, in agreement with a previous report [16, 24]. The open probability (Po) of the hBK channel showed a voltage dependency. Po was higher at negative voltages on the side of the recording solution, that is, the side of the probe was at positive voltages (Fig. 3b). As it is known that the Po of the hBK channel is higher when the intracellular domain is at more positive voltages [24], these results indicate that the hBK channel was incorporated into the lipid bilayer membrane such that its intracellular domain side was on the probe side and supported that the channels immobilized on the probe were incorporated into the membrane.

Using this technique, we can align the orientation of the hBK channel. Aligning the channel orientation is difficult using conventional artificial lipid bilayer recording techniques when ion channels in liposomes are not all oriented in the same direction. The orientation uniformity is useful for drug screening because drugs that act only on the extracellular or intracellular sides of the ion channel can be identified. It is possible to identify drugs that act on the extracellular side of the hBK channel by immobilizing the C-terminus, which is located on the intracellular side, on the probe, and drugs that act on the intracellular side of the channel by immobilizing the N-terminus, which is located on the extracellular side, on the probe.

## Conclusion

In this study, we improved our previously developed technique to efficiently measure the current of the hBK channel. Using a gold probe on which liposomes containing hBK channels were immobilized, the channels were efficiently incorporated into the lipid bilayer membrane and the measured current showed the same characteristics as the current measured using conventional artificial lipid bilayer recording techniques. The developed technique would be potentially applicable to any other ion channel that is difficult to incorporate into the membrane. In addition, this technique allowed us to align the channel orientation in the lipid bilayer membrane, which is difficult with conventional methods when ion channels in liposomes are not uniformly oriented. This advantage would be useful for selecting drugs that act only on the extracellular or intracellular sides of the channels. In the future, we plan to develop a high-throughput system for ion channels using this technique.

## Supplementary Information

Below is the link to the electronic supplementary material.

Supplementary file1 (DOCX 91 KB)

## Data Availability

The data of this study are available from the corresponding author upon reasonable request.
