# Supplementary material for: Efficient single-channel current measurements of the human BK channel using a liposome-immobilized gold probe
Source: Anal Sci. 2024 Dec 20;41(4):329–34. doi: 10.1007/s44211-024-00707-3 (PMC11937041; doi:10.1007/s44211-024-00707-3)
Supplement: Supplementary file 1 — Supplementary file1 (DOCX 91 KB) [file 44211_2024_707_MOESM1_ESM.docx]

**Supplementary Information**

Title:

**Efficient single-channel current measurements of the human BK channel using a liposome-immobilized gold probe**

Journal name:

**Analytical Sciences**

Author names:

**Minako Hirano*****, Mami Asakura, Toru Ide**

Affiliation

**Graduate School of Interdisciplinary Science and Engineering in Health Systems, Okayama University, 3-1-1 Tsushima-naka, Kita-ku, Okayama-shi, Okayama 700-8530, Japan**

E-mail address of the corresponding author:

**hirano37@okayama-u.ac.jp (Minako Hirano)**

**
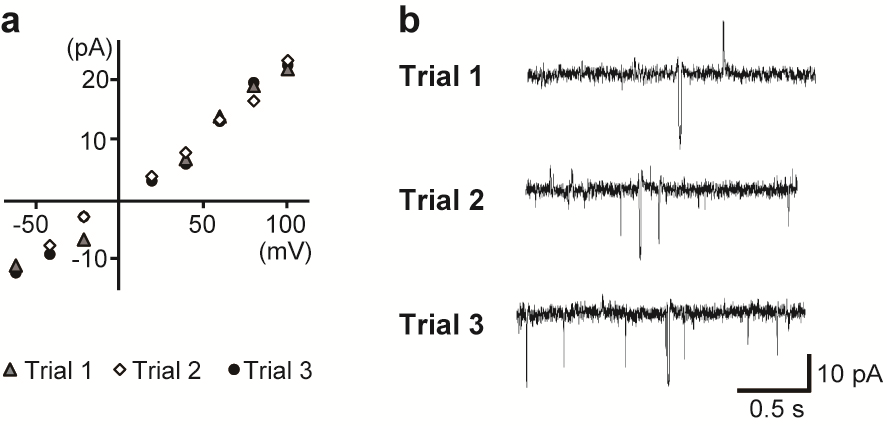
**

**Figure S1.** Single-channel current recordings of the hBK channel measured multiple times using the probe with hBK channel-containing liposomes ("Probe-hBK-Liposome"). (a) Current-voltage relationships of the hBK channel measured in trial 1, 2, and 3. (b) Typical single-channel current traces of the hBK channel at +80 mV from each measurement.
